# Repressive Chromatin in *Caenorhabditis elegans*: Establishment, Composition, and Function

**DOI:** 10.1534/genetics.117.300386

**Published:** 2018-02-25

**Authors:** Julie Ahringer, Susan M. Gasser

**Affiliations:** * The Gurdon Institute, University of Cambridge CB2 1QN, United Kingdom; † Department of Genetics, University of Cambridge CB2 1QN, United Kingdom; ‡ Friedrich Miescher Institute for Biomedical Research (FMI), 4058 Basel, Switzerland, and; § Faculty of Natural Sciences, University of Basel, 4056, Switzerland

**Keywords:** *C. elegans*, chromatin, heterochromatin, histone methylation, H3K9me, H3K27me, WormBook

## Abstract

Chromatin is organized and compacted in the nucleus through the association of histones and other proteins, which together control genomic activity. Two broad types of chromatin can be distinguished: euchromatin, which is generally transcriptionally active, and heterochromatin, which is repressed. Here we examine the current state of our understanding of repressed chromatin in *Caenorhabditis elegans*, focusing on roles of histone modifications associated with repression, such as methylation of histone H3 lysine 9 (H3K9me2/3) or the Polycomb Repressive Complex 2 (MES-2/3/6)-deposited modification H3K27me3, and on proteins that recognize these modifications. Proteins involved in chromatin repression are important for development, and have demonstrated roles in nuclear organization, repetitive element silencing, genome integrity, and the regulation of euchromatin. Additionally, chromatin factors participate in repression with small RNA pathways. Recent findings shed light on heterochromatin function and regulation in *C. elegans*, and should inform our understanding of repressed chromatin in other animals.

EUKARYOTIC DNA is organized and compacted in the nucleus through its association with histones and nonhistone proteins, forming a complex called chromatin. The N-terminal tails of all histones, as well as the C-terminal tails of histones H2A and H2B, are subject to post-translational modifications that selectively impact many aspects of nuclear function. The most common histone modifications include methylation, acetylation, ubiquitination, sumoylation, and phosphorylation.

Two broad classes of chromatin, euchromatin and heterochromatin, can be distinguished based on protein composition, characteristic post-translational modifications on histones, and transcriptional activity. Euchromatin is either potentially or actively transcribed, and is enriched for RNA polymerase, histone tail acetylation, and trimethylation on histone H3 lysines 4 and 36 (H3K4me3 and H3K36me3). In contrast, heterochromatin tends to be transcriptionally repressed and is associated with histone methylations such as H3K9me3 or the Polycomb-deposited modification H3K27me3. The proteins that recognize these modified histones and the association of heterochromatin with the nuclear envelope help hold chromatin in a compact conformation, and promote the spread of the repressed chromatin state (Eskeland *et al.* 2007; Towbin *et al.* 2010; Elgin and Reuter 2013; Simon and Kingston 2013; Wiles and Selker 2016). Despite its heritable nature, heterochromatin is nonetheless dynamically regulated. Moreover, its tendency to aggregate and create repressive subnuclear compartments means that it can also indirectly influence the organization of euchromatin and gene expression (Francastel *et al.* 2000; Sexton *et al.* 2007).

Although there are many types of repressed chromatin, reflecting a range of modifications and ligands, heterochromatin was traditionally split into two classes that largely reflect the properties of the underlying DNA. On one hand, constitutive heterochromatin covered regions of the genome that are repeat rich and gene poor, and that are kept in a silent state throughout cell division and cell differentiation by H3K9me2/3 and its ligand, Heterochromatin Protein 1 (HP1) (Eissenberg and Elgin 2014; Saksouk *et al.* 2015; Wang *et al.* 2016). Facultative heterochromatin, on the other hand, encompasses genes that are potentially active, such as those with spatial, temporal, or other types of context-specific expression. Its hallmark is H3K27me3, which is deposited by Polycomb Repressive Complex 2 (PRC2), and which defines a pathway that maintains transcriptional repression (Wiles and Selker 2016).

Recent findings suggest that these classical distinctions are inadequate to describe the complexity of heterochromatin types. For instance, chromatin immunoprecipitation (ChIP) analyses in *Caenorhabditis elegans* have shown that much of the H3K9me3-marked chromatin coincides with H3K27me3 (Liu *et al.* 2011). In mammals, H3K9me3 and H3K27me3 are negatively correlated when scored for coincidence on the same histone tail, yet some genomic regions carry both modifications, as detected by ChIP. Additionally, cooperation between H3K9 and H3K27 methylation in heterochromatin formation has been reported (Hawkins *et al.* 2010; Boros *et al.* 2014; Schwammle *et al.* 2016). Finally, H3K23me2 has been reported to coincide independently with K9 and K27 methylation on histone H3 tails in *C. elegans* (Vandamme *et al.* 2015; Sidoli *et al.* 2016), much like the trimethylation of H4K20 in mammals, which accumulates on both facultative and constitutive heterochromatin during cellular senescence (Nelson *et al.* 2016). Here, we focus on histone H3K9 and H3K27 methylations, as they are the best-understood heterochromatin marks, and are involved in genetically distinct but highly conserved pathways of transcriptional repression.

## Histone Methyltransferases and Recognition of Modifications

The enzymes responsible for histone lysine methylation are called histone methyltransferases (HMTs). HMTs typically contain a conserved catalytic domain called SET, which stems from **S**u(var)3-9, **E**nhancer of zeste, and **T**rithorax, the first HMTs known to carry this domain (Tschiersch *et al.* 1994). The SET domain contains a S-adenosylmethionine (SAM)-binding site and a catalytic center (Yeates 2002). The *C. elegans* genome encodes 38 SET domain-containing, putative HMTs (Andersen and Horvitz 2007). A loss-of-function mutant has been isolated for 30 of these, of which five are essential for viability (Andersen and Horvitz 2007; Ni *et al.* 2012). The preferred substrates of most HMTs have not yet been identified in *C. elegans*, apart from: SET-25 and MET-2/SETDB1, which target H3K9 (Bessler *et al.* 2010; Towbin *et al.* 2012); MES-2/EZH2, which modifies H3K27 (Bender *et al.* 2004); SET-1 and SET-4, which modify H4K20 (Vielle *et al.* 2012); and MET-1 and MES-4, which are responsible for H3K36 methylation (Bender *et al.* 2006; Furuhashi and Kelly 2010; Rechtsteiner *et al.* 2010). One cannot exclude the possibility that these HMTs (summarized in Table 1 and Table 2) modify lysines in other proteins as well.

**Table 1:** Chromatin proteins discussed in this review

Protein	Ortholog	Domains	Description
Histone methyltransferases			
MET-2	SETDB1	SET, MBD, Pre-SET, Post-SET	H3K9me1/2 HMT
SET-25	G9a/SUV39 SET)	SET Post-SET	H3K9me1/2/3 HMT
SET-32		SET	Putative H3K9me3 HMT
SET-1	PR-Set7/SETD8	SET	H4K20me1 HMT
SET-4	SET4-20	SET	H4K20me2/3 HMT
MES-4	NSD1-3	SET, Post-SET, PHD	H3K36me2/3 HMT
MET-1	SET-2	SET	H3K36me3 HMT
PRC2-like complex			
MES-2	EZH2	SET, CXC	PRC2 complex/H3K27 HMT
MES-3			component of PRC2 complex
MES-6	ESC/EED	WD40	Component of PRC2 complex
Histone demethylases			
SPR-5	KDM1A, LSD1	SWRIM, amino oxidase	H3K4 demethylase
JMJD-1.2	PHF8	PHD, JmjC	H3K9me2/H3K27me2 and H3K23 demethylase
JMJD-3.1	KDM6B, JMJD3	JmjC	H3K27me2/3 demethylase
UTX-1	KDM6A, UTX	JmjC, TPR repeat	H3K27me2/3 demethylase
Heterochromatin-associated proteins			
HPL-1	HP1	Chromo, chromoshadow	Binds HIS-24/H1K14me1 *in vitro*
HPL-2	HP1	Chromo, chromoshadow	Binds H3K9me1/2/3 *in vitro*
CEC-3		Chromo	Binds H3K9me1/2/3 *in vitro*
CEC-4		Chromo	Binds H3K9me1/2/3 *in vitro* and *in vivo*
LIN-61		MBT	Binds H3K9me1/2/3 *in vitro*
LIN-13		C2H2 and RING/FYVE/PHD-type zinc fingers	In complex with HPL-2 and LIN-61
LET-418	Mi-2, CHD3	PHD, chromo, Helicase_C, SNF2_N, CHDCT2	Nucleosome remodelling component of NuRD and Mec complexes
Nuclear lamina proteins			
LMN-1	Lamin A and B	Coiled-coil, Ig, and CAAX box	Nuclear intermediate filament protein
LEM-2	MAN1	LEM, Man1-Src1p-C-term	Lamin-binding INM protein
EMR-1	Emerin	LEM, Man1-Src1p-C-term	Lamin-binding INM protein
SUN-1	SUN1,2,3,5	SUN	INM-spanning protein that binds KASH domain
UNC-84	SUN1,2,3,5	SUN	INM-spanning protein that binds KASH domain
BAF-1	BANF1, BAF	BAF	dsDNA and lamin and LEM domain ligand
Small RNA pathway proteins			
PRG-1	Piwi	PAZ, Piwi	piRNA pathway argonaute
NRDE-1			Novel nuclear RNAi factor
NRDE-2	NRDE2	NRDE-2	Nuclear RNAi factor, interacts with NRDE-3
NRDE-3		PAZ, Piwi	Somatic nuclear RNAi argonaute
NRDE-4			Novel nuclear RNAi factor
HRDE-1		PAZ, Piwi	Germ line nuclear RNAi argonaute
MORC-1	MORC1, MORC2	HATPase_c	Nuclear RNAi pathway effector

The *C. elegans* genome contains 38 SET domain proteins, 6 amino oxidase-type putative histone demethylases, 14 jmjC domain proteins, 67 putative histone mark readers (bearing either a chromodomain, Tudor, MBT, PHD, or WD-40 domain), 27 argonaute domain proteins, and an as yet undetermined number of nuclear lamina-associated proteins [for a more complete survey of nuclear envelope proteins see Dobrzynska *et al.* (2016)]. See text for references and types of data supporting these definitions. In the case of HMTs and histone mark readers, only a few are supported by mass spectrometric data, point mutations within the active domain, and/or an exhaustive analysis of potential ligands. Data based on genetic phenotypes and colocalization should be considered suggestive but not conclusive. SET, Su(var)3-9, Enhancer of zeste, and Trithorax; HMT, histone methytransferases; PHD, plant homeodomain; TPR, tetratricopeptide repeat; MBT, malignant brain tumor; RING; FYVE, Fab 1, YOTB, Vac 1 and EEA1; LEM, Lamin and Emerin; INM, inner nuclear membrane; SUN, Sad1/UNC-84-homology; KASH, Klarsicht/ANC-1/Syne-1 homology; BAF, barrier-to-autointegration factor; PAZ, Piwi Argonaut and Zwille; RNAi, RNA interference; piRNA, piwi-interacting RNA.

**Table 2:** Loss-of-function phenotypes of genes discussed in this review

Loss-of-function phenotypes											
Gene	Superficially wild-type	Sterile	Lethal	Increased germ line apoptosis	L1 arrest*^a^*	synMuv	Increased mutation rate	Somatic expression of germ line genes	Heterochromatic array desilencing	Other phenotypes	References
Histone methyltransferases											
* met-2*	x	mrt		x		x		x	Yes	Satellite repeat transcription	Poulin *et al.* (2005), Andersen and Horvitz (2007), Bessler *et al.* (2010), Koester-Eiserfunke and Fischle (2011), Towbin *et al.* (2012), Wu *et al.* (2012), Zeller *et al.* (2016), J. Padeken, P. Zeller, and S. M. Gasser, (unpublished results)
* set-25*	x						x		Yes	Retrotransposon expression	Pothof *et al.* (2003), Andersen and Horvitz (2007), Towbin *et al.* (2012), Zeller *et al.* (2016), McMurchy *et al.* (2017), J. Padeken, P. Zeller, and S. M. Gasser, (unpublished results)
* set-32*	x	mrt								Nuclear RNAi and piRNA pathway effector	Andersen and Horvitz (2007), Ashe *et al.* (2012), Kalinava *et al.* (2017), Spracklin *et al.* (2017)
* set-1*		ste								Dosage compensation defect	Vielle *et al.* (2012), Wells *et al.* (2012), Kramer *et al.* (2016)
* set-4*	x										Vielle *et al.* (2012), Wells *et al.* (2012), Kramer *et al.* (2016)
* mes-4*		mes							Yes	synMuv suppressor	Capowski *et al.* (1991), Cui *et al.* (2006), Towbin *et al.* (2012)
* met-1*	x					x			No		Andersen and Horvitz (2007), Towbin *et al.* (2012), Wu *et al.* (2012)
* met-2 set-25*	x	mrt		x			x		Yes	Loss of heterochromatin anchoring; repeat element expression	Towbin *et al.* (2012), Zeller *et al.* (2016), McMurchy *et al.* (2017)
PRC2-like complex											
* mes-2*		mes								Yes	Capowski *et al.* (1991), Towbin *et al.* (2012)
* mes-3*		mes								Yes	Capowski *et al.* (1991), Towbin *et al.* (2012)
* mes-6*		mes								Yes	Capowski *et al.* (1991), Towbin *et al.* (2012)
Histone demethylases											
* spr-5*	x	mrt									Katz *et al.* (2009), Greer *et al.* (2016)
* jmjd-1.2*	x									Locomotion; mitochondrial stress-induced longevity defect; DNA repair defect	Lee *et al.* (2015), Merkwirth *et al.* (2016), Riveiro *et al.* (2017)
* jmjd-3.1*										Abnormal gonad development; transdifferentiation defect	Agger *et al.* (2007), Zuryn *et al.* (2014)
* utx-1*		ste*^b^*				x					Wang *et al.* (2010), Vandamme *et al.* (2012)
Heterochromatin-associated proteins											
* hpl-1*	x										Schott *et al.* (2006)
* hpl-2*		ste*^c^*		x	x	x		x	Yes		Capowski *et al.* (1991), Couteau *et al.* (2002), Wang *et al.* (2005), Schott *et al.* (2006), Petrella *et al.* (2011), Towbin *et al.* (2012), Wu *et al.* (2012), McMurchy *et al.* (2017)
* cec-3*	x									Suppresses *spr-5* Transgenerational sterility; *unc-4* ectopic expression	Zheng *et al.* (2013), Greer *et al.* (2014)
* cec-4*	x								No	Loss of perinuclear chromatin anchoring	Gonzalez-Sandoval *et al.* (2015)
* lin-61*	x			x	x	x	x	x	Yes	Compromised homology-driven repair	Pothof *et al.* (2003), Poulin *et al.* (2005), Harrison *et al.* (2007), Koester-Eiserfunke and Fischle (2011), Petrella *et al.* (2011), Towbin *et al.* (2012), Wu *et al.* (2012), Johnson *et al.* (2013), McMurchy *et al.* (2017)
* lin-13*		ste		x	x	x		x	Yes		Ferguson and Horvitz (1985), Melendez and Greenwald (2000), Wang *et al.* (2005), Petrella *et al.* (2011), Wu *et al.* (2012), McMurchy *et al.* (2017)
* let-418*		ste			x	x		x	No		Solari and Ahringer (2000), von Zelewsky *et al.* (2000), Unhavaithaya *et al.* (2002), Passannante *et al.* (2010), Wu *et al.* (2012)
Small RNA pathway proteins											
* prg-1*	x	mrt		x							Batista *et al.* (2008), Das *et al.* (2008), Simon *et al.* (2014), McMurchy *et al.* (2017)
* nrde-1*	x	mrt							Yes		Burkhart *et al.* (2011), Buckley *et al.* (2012), J. Padeken, P. Zeller, and S. M. Gasser, (unpublished results)
* nrde-2*	x	mrt									Guang *et al.* (2010), Buckley *et al.* (2012)
* nrde-3*	x	mrt							Yes		Guang *et al.* (2008), J. Padeken, P. Zeller, and S. M. Gasser, (unpublished results)
* nrde-4*	x	mrt									Burkhart *et al.* (2011), Buckley *et al.* (2012), J. Padeken, P. Zeller and S. M. Gasser, (unpublished results)
* hrde-1*	x	mrt									Ashe *et al.* (2012), Buckley *et al.* (2012), Luteijn *et al.* (2012), Shirayama *et al.* (2012)
* morc-1*	x	mrt									Spracklin *et al.* (2017), Weiser *et al.* (2017)

Common phenotypes observed in chromatin-modulating mutants are listed. Phenotypes listed are not comprehensive and were not assessed in all mutants. synMuv, synthetic multivulval; mrt, mortal germ line (transgenerationally sterile); RNAi, RNA interference; piRNA, piwi-interacting RNA; ste, zygotic sterile; mes, maternal-effect sterile.

a Some mutants undergo L1 arrest only at high temperature (26°).

b Homozygotes are nearly sterile, and the few embryos produced die.

c Null mutant is sterile at 25°.

d Co-RNAi or double mutant of *lem-2* and *emr-1* is lethal.

Histone modifications can directly alter nucleosome–nucleosome or nucleosome–DNA interactions by changing the charge of the highly basic histone tail or disrupting contact sites between DNA and the nucleosomal core particle. Alternatively, specific histone modifications can create binding sites for proteins that specifically recognize a given modified amino acid. These “readers” of post-translational histone modifications can in turn alter the chromatin compaction state, or recruit additional transcriptional regulators or chromatin-modifying enzymes. A growing list of structural motifs have been shown to recognize modified histones, the most common being Bromo, Chromo, Tudor, malignant brain tumour (MBT), plant homeodomain (PHD)PHD, WD40 repeat (∼40 amino acid terminating in Trp-Asp) 14-3-3, and BRCT (BRCA1 C Terminus) domains (Taverna *et al.* 2007). *C. elegans* has 67 proteins containing such domains, which are predicted to be readers of histone modifications (Towbin *et al.* 2012; Gonzalez-Sandoval *et al.* 2015). Histone H3 tail methylations alone are known to be recognized by Chromo, MBT, PWWP (Pro-TrpTrp-Pro motif) or Tudor domains, as well as by specialized WD40 repeat structures (Margueron *et al.* 2009; Xu *et al.* 2010; Khorasanizadeh 2011).

## The C. elegans Genome and the Distribution of Heterochromatin

The 100-Mbp genome of *C. elegans* is separated into five autosomes and an X chromosome (*C. elegans* Sequencing Consortium 1998). Hermaphrodites are diploid for all six chromosomes, whereas males have five pairs of autosomes and only a single X chromosome. The regulation and characteristics of the autosomal chromosomes differ from the X chromosome, in part due to dosage compensation in the soma, which represses gene expression approximately twofold on the two hermaphrodite X chromosomes to match expression on the single male X. Here, we do not cover dosage compensation, but refer the reader to reviews of this subject (Meyer 2010; Strome *et al.* 2014).

**Figure 1:**
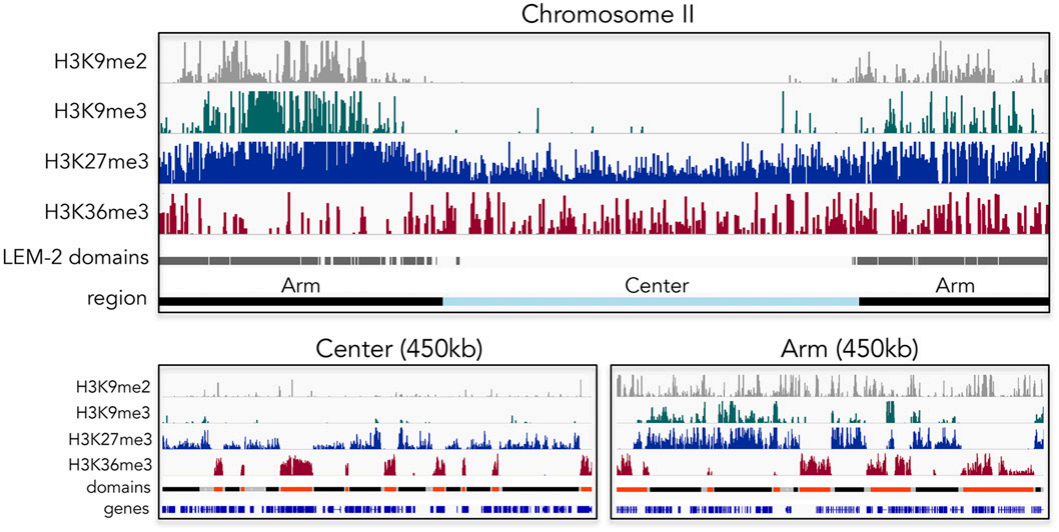
Histone modifications associated with heterochromatin and genome domains. Top: pattern of histone modifications associated with heterochromatin (H3K9me2, H3K9me3, and H3K27me3), H3K36me3, and LEM-2 domains (indicating nuclear lamina association) in embryos across *C. elegans* chromosome II. The majority of H3K9me marks and LEM-2 domains are found on the chromosome arms. H3K27me3 levels are higher on arm regions compared to the center. H3K36me3 shows a more uniform pattern, with a slight enrichment in the central region. Bottom left: 450-kb segment of a central region showing low H3K9 methylation, and anticorrelated domains marked by H3K27me3 and H3K36me3. H3K36me3 is found in active chromatin domains (orange) and H3K27me3 in regulated chromatin domains (black), which are separated by border regions (gray). Bottom right: 450-kb segment of an arm region. The distributions of H3K9me3 and H3K27me3 are largely similar to each other, but differ from H3K9me2. Patterns of H3K36me3 and H3K27me3 and chromatin domains are similar to those in central regions.

Like most nematodes, *C. elegans* chromosomes are holocentric (Albertson and Thomson 1982; Maddox *et al.* 2004). That is, centromere activity is distributed along chromosomes at sites of incorporation of a variant histone H3 CENP-A instead of being focused in a single, heritable centromeric site (Gassmann *et al.* 2012). The distal arms and central regions of the five autosomes have different characteristics. Most meiotic recombination occurs in the distal arm regions, where genes are on average longer and have larger introns. Genes in central regions are generally more highly expressed and show higher evolutionary conservation (*C. elegans* Sequencing Consortium 1998). These differences are reflected in the distributions of chromatin modifications. For example, average levels of modifications associated with gene activity are higher in central regions (Liu *et al.* 2011). Di- and trimethylation of H3K9, hallmarks of constitutive heterochromatin, are both predominantly found on distal chromosome arms, as is H3K9me1, although they have distinct distributions, reflecting distinct modes of recruitment of the relevant HMTs (Gu and Fire 2010; Liu *et al.* 2011) (J. Padeken, P. Zeller, and S. M. Gasser, unpublished results; Figure 1). Nonetheless, the distal arms are not devoid of transcribed genes, and those found interspersed among heterochromatic domains carry the same histone modifications as actively transcribed genes in central chromosomal domains (Liu *et al.* 2011).

In contrast to constitutive heterochromatin, facultative heterochromatin carries H3K27 trimethylation, the most abundant histone methylation mark as measured by mass spectrometry in *C. elegans*. H3K27me3 is found on 67% of histones in embryos (Vandamme *et al.* 2015), and maps both to distal arms and central chromosome regions (Liu *et al.* 2011; Figure 1). Profiling early embryos and L3 larvae, levels were found to be higher on chromosome arms and higher on the X chromosome than on autosomes. On distal chromosome arms, H3K27me3 often colocalizes by ChIP sequencing with H3K9me3, as it does on large integrated arrays of transgenes, whereas in central regions of autosomes H3K27me3 is found without H3K9me3 (Meister *et al.* 2010; Liu *et al.* 2011) (Figure 1). Consistent with a role in transcriptional repression, the genome-wide distribution of H3K27me3 is anticorrelated with RNA levels and the presence of RNA polymerase (Liu *et al.* 2011).

While *C. elegans* has the repressive histone methyl marks and the ligands that are present in other organisms, it lacks 5-methyl cytosine on DNA and the 5meC-binding proteins that repress transcription in vertebrates. A very low level of adenine N(6)-methylation (6mA) has been reported on DNA in *C. elegans* (Greer *et al.* 2015), yet given that 6mA is the most abundant RNA modification, and no dedicated adenine methyltransferase for DNA has been identified, it is unclear whether 6mA-DNA has any physiological significance.

## The Spatial Organization of Repetitive DNA

Roughly 20% of the *C. elegans* genome is repetitive DNA (Hillier *et al.* 2008; Hubley *et al.* 2016), including tandem repeats and sequences derived from DNA or RNA transposons. These repetitive elements are enriched in the distal arm regions, and are generally marked by nucleosomes bearing H3K9me2 and/or H3K9me3 (*C. elegans* Sequencing Consortium 1998; Gerstein *et al.* 2010; Zeller *et al.* 2016; McMurchy *et al.* 2017). Among repetitive sequences, those derived from DNA transposons are particularly abundant (covering 12.6% of the genome). Some DNA transposons can be activated, yet only a small minority of elements encode full-length transposases (Bessereau 2006; Hubley *et al.* 2016). LTR-containing and LTR-free sequences derived from RNA transposons (retrotransposons) cover ∼1% of the genome, but there are few full-length elements, and none appear to be active under wild-type conditions (Bessereau 2006; Hubley *et al.* 2016).

In organisms with localized centromeres, pericentric domains contain large arrays of tandem satellite repeats, which can occupy up to 1 Mbp in vertebrates (Plohl *et al.* 2008). Difficulties in sequencing and mapping these regions unambiguously have meant that the exact extent and composition of repetitive domains are unclear for most vertebrate genomes, yet pericentric satellite sequences are estimated to account for 5–10% of the human genome (Schueler and Sullivan 2006).

The *C. elegans* genome contains smaller arrays of tandem simple repeats (microsatellites of 1–6 bp), as well as minisatellite repeats (10–200 bp) that are distributed rather than clustered in large head-to-tail arrays (Subirana *et al.* 2015; Hubley *et al.* 2016). This is reminiscent of the dispersed nature of *C. elegans* centromeres. The shortness of the repeat clusters has allowed for high-quality sequencing of nearly all *C. elegans* repetitive DNA (*C. elegans* Sequencing Consortium 1998; Hillier *et al.* 2005). Additionally, 71% of the repeats are uniquely mappable with 50-bp single-end reads. Interestingly, several microsatellite families have been shown to be enriched in CENP-A, suggesting that they may be associated with centromere function (Subirana *et al.* 2015), although centromere function in worms is independent of H3K9 methylation (Towbin *et al.* 2012; Ho *et al.* 2014; Garrigues *et al.* 2015; Zeller *et al.* 2016).

## The Nuclear Lamina and Chromatin Association

The mapping of chromatin regions associated with proteins of the nuclear lamina has shown that the central and distal arm regions of *C. elegans* chromosomes have distinct distributions with respect to the nuclear envelope (Ikegami *et al.* 2010; Towbin *et al.* 2012; Gonzalez-Aguilera *et al.* 2014). *C. elegans* expresses a single lamin protein (LMN-1), that forms a stable meshwork underlying the nucleoplasmic side of the nuclear envelope together with a number of well-characterized lamin-associated proteins (Dobrzynska *et al.* 2016). These lamin-associated factors include the LEM domain proteins LEM-2 (MAN1) and EMR-1 (Emerin), the SUN domain proteins UNC-84 and SUN-1, the accessory protein BAF, and four KASH domain proteins that form a bridge to the cytoskeleton (Bank and Gruenbaum 2011, see Table 1 for abbreviations). While this is undoubtedly a nonexhaustive list, this set of proteins and functions is conserved across animal species.

The genome-wide mapping profiles of LEM-2, LMN-1, and EMR-1 all show strong enrichment on distal chromosome arms, indicating that these regions are associated with the nuclear envelope (Ikegami *et al.* 2010; Towbin *et al.* 2012; Gonzalez-Aguilera *et al.* 2014; Gonzalez-Sandoval *et al.* 2015). A detailed analysis of LEM-2 binding showed that these domains are not continuous along the chromosome arms, but are interspersed with gaps that bear expressed genes (Ikegami *et al.* 2010), which are thought to extend inwards from the peripheral lamin- or LEM-2-associated domains. The LEM-2-associated domains are enriched for H3K9-methylated histones, and their perinuclear positioning is largely dependent on this mark, which is specifically recognized by a nuclear envelope-associated chromodomain protein, CEC-4 (Towbin *et al.* 2012; Gonzalez-Sandoval *et al.* 2015). The LEM-2-bound regions are not always transcriptionally silent, and those with gene expression are enriched for the HP1 homolog HPL-2 (Garrigues *et al.* 2015).

## Phenotypes of Chromatin Repressor Mutants

In *C. elegans*, the development of the vulva has proven to be a powerful system to identify regulators of cell fate, as it is dispensable for survival and defects are easily observable (Horvitz and Sternberg 1991). Many genes with presumed roles in chromatin repression, including those encoding proteins that generate or bind methylated H3K9, were originally identified based on their synthetic multivulval (synMuv) phenotype (Fay and Yochem 2007). Single mutants of synMuv genes have a normal vulva, but double mutants between synMuv genes of different genetic classes develop extra vulvae, arguing for a partial functional redundancy between synMuv gene classes. The synMuv genes were found to encode proteins with a wide range of chromatin-modifying activities, including enzymes that deposit or remove methylation and acetylation on histone tails, ligands for the methylated residues, and nucleosome remodelers. Interestingly, ectopic vulval development in synMuv mutants was shown to be due to a failure to repress expression of a single gene, *lin-3*/EGF, in the epidermis (Cui *et al.* 2006). Of note, most synMuv genetic interactions occur between genes that encode different biochemical activities (*e.g.*, between an acetyltransferase and a deacetylase). This functional redundancy reflects the inherent complexity found in chromatin regulatory mechanisms, and argues for redundancy in repression mechanisms. Although synMuv genes were identified based on their roles in the repression of vulval development, most are widely expressed, and the single mutants often show pleiotropic developmental defects and genetic interactions in nonvulval processes. Common phenotypes among them are impaired fertility, altered regulation of repetitive transgenes, L1 arrest at high temperature, and ectopic expression of germ line genes in somatic tissues (Table 2, references therein). We refer readers to chapters on developmental roles of chromatin factors and the synMuv genes for more detailed information (Cui and Han 2007; Fay and Yochem 2007).

## Histone H3K9 Methyltransferases

Over the years, a number of *C. elegans* genes have been postulated to encode HMTs that target H3K9me [*e.g.*, MET-2, SET-9, SET-26, SET-25, and SET-32; (Bessler *et al.* 2010; Towbin *et al.* 2012; Greer *et al.* 2014; Ni *et al.* 2016; Kalinava *et al.* 2017)]. However, it has been shown recently that the vast majority of H3K9me1, me2, and me3 depend on two key enzymes: MET-2, which is able to deposit me1 and me2 on H3K9, and SET-25, the major, if not only, HMT that deposits H3K9me3 in somatic cells of larvae and embryos (Towbin *et al.* 2012). In a double knockout for these two SET domain genes, embryos and L1 stage larvae lacked all detectable H3K9me when analyzed by mass spectrometry (Towbin *et al.* 2012; Garrigues *et al.* 2015). Moreover, immunofluorescence (IF) of embryos, L2 larvae, and dissected gonads of the double mutant showed no H3K9me signal (Towbin *et al.* 2012; Ho *et al.* 2014; Garrigues *et al.* 2015; Zeller *et al.* 2016). Thus, if other H3K9-modifying HMTs exist in worms, either they are expressed under very select conditions or in very few cells, or else their activity requires SET-25 or MET-2. Based on the lower limit of detection by the mass spectroscopy method used, we estimate that < 5% of embryonic histone H3K9 methylation is retained in a *set-25met-2* double mutant.

SET-32, a germ line-specific protein whose SET domain has weak homology with EHMT1/G9a of humans, may provide a low level of H3K9me3 methylation in the germ line. Two recent studies found that *set-32* mutants are transgenerationally sterile and had reduced H3K9me3 on nuclear RNA interference (RNAi) targets in young adults (Kalinava *et al.* 2017; Spracklin *et al.* 2017). However, at most genomic locations, H3K9me3 levels were as low in *met-2set-25* double as in *met-2set-25*; *set-32* triple mutants (Kalinava *et al.* 2017), confirming previous studies reporting loss of detectable H3K9 methylation in *met-2set-25* mutants, both in gonads and somatic cells (Towbin *et al.* 2012; Garrigues *et al.* 2015; Zeller *et al.* 2016). MES-2, the EZH2-like H3K27 methyltransferase, was also initially suggested to modify H3K9 in the germ line (Bessler *et al.* 2010). However, the alterations detected were likely due to antibody cross-reactivity between methylated H3K27 and H3K9. Using validated H3K9me3 antibodies, *mes-2* mutant germ lines have normal levels of H3K9me3 staining, while *met-2set-25* double-mutant germ lines have none (Ho *et al.* 2014; Zeller *et al.* 2016).

MET-2, the *C. elegans* homolog of mammalian SETDB1/ESET, was first described as a potential transcriptional repressor based on its synMuv phenotype (Poulin *et al.* 2005; Andersen and Horvitz 2007). SET-25 is less conserved, yet its catalytic SET domain shares 28.8% identity and 44.6% similarity in protein sequence with mammalian EHMT1/G9a, as well as 27.9% identity and 45.7% similarity with Suv39h1/2, although SET-25 lacks both the chromodomain found in Suv39h and the Ankyrin repeats present in G9a. Because the relative abundance of other common H3 tail methylation marks did not change in the *set-25met-2* mutant (Towbin *et al.* 2012), it is likely that these enzymes are specific for histone H3K9. However, one cannot exclude the possibility that the HMTs modify nonhistone targets as well.

Using a fluorescent heterochromatin reporter that allows the quantification of transcriptional silencing, nuclear position, and nucleosome modifications, it was shown that MET-2 works together with SET-25 to silence constitutively expressed promoters, and to tether silent transgene arrays and endogenous repeat sequences at the nuclear envelope (Towbin *et al.* 2012). SET-25 was shown to be essential for all H3K9me3 in embryos and L1 larvae, and to maintain ∼20% of wild-type levels of H3K9me1 and me2 in *met-2* mutants. MET-2, on the other hand, is the main H3K9 mono- and dimethyltransferase, and it can compensate for SET-25 to maintain wild-type levels of H3K9me1 and me2 in *set-25* embryos and L1 larvae (Towbin *et al.* 2012). Dissecting their individual contributions to chromatin localization using null alleles, it was shown that MET-2 is sufficient to confer anchoring of integrated transgene arrays and endogenous heterochromatin at the nuclear envelope, while SET-25 activity in a *met-2* mutant was sufficient to anchor the integrated transgene arrays only.

There is evidence that both MET-2 and SET-25 associate with their own enzymatic products in the nucleus. Importantly, whereas a highly overexpressed MET-2-GFP fusion protein is primarily cytoplasmic (Towbin *et al.*, 2012), and a MET-2-mCherry fusion expressed as a single-copy gene from the *met-2* promoter gives a spotty nuclear signal (M. Guidi and S. M. Gasser, unpublished results). Moreover, the nuclear MET-2 ChIP pattern in adults is very similar to that of its product H3K9me2 (McMurchy *et al.* 2017). SET-25 is exclusively nuclear, and it binds to H3K9me3 in a SET domain-independent manner, marking heterochromatin- and H3K9me3-enriched foci (Towbin *et al.* 2012). In other words, once SET-25 trimethylates H3K9, it either recognizes its product or else binds another reader that recognizes this mark, such that it remains associated with the chromatin that it modified. The association of SET-25 with silent chromatin means that it can act to extend methylation to nearby histone H3 tails, ensuring the spread or potentially self-maintenance of heterochromatic domains.

H3K23me2 was recently shown to be strongly associated with H3K27- and H3K9-methylated heterochromatin in *C. elegans* (Vandamme *et al.* 2015; Sidoli *et al.* 2016). Methylated forms of H3K23 are also found in mouse, where the levels of modification seem to correlate with H3K27 methylation in a Suz12 (PRC2) mutant (Schwammle *et al.* 2016). Hints regarding its function are suggested by the affinity of the mammalian HP1β chromodomain for H3K23me1/2/3 *in vitro* (Liu *et al.* 2010) and the apparent affinity of *C. elegans* HPL-1 for H3K23me1/2 in binding assays *in vitro* (Vandamme *et al.* 2015). This association suggests that H3K23 methylation may function in transcriptional repression, although functional analysis requires identification of the HMT and demethylase that are responsible for the deposition and removal, respectively, of this mark. Interestingly, the H3K9me2 and H3K27me2 histone demethylase JMJD-1.2 appears to act on H3K23me2, as *jmjd-1.2* mutants show increased H3K23me2 by IF and recombinant JMJD-1.2 can demethylate H3K23me2 *in vitro* (Lin *et al.* 2010). Intriguingly, this interaction appears to be conserved in mouse (Liu *et al.* 2010).

## H3K9me Readers

Given that lysine methylation does not mask the positive charge of the side chain, but instead deposits a bulky adduct, this modification is thought to work primarily through the recruitment of specific ligands or readers. The prototype H3K9me reader is *Drosophila* HP1a, which has been shown to bind H3K9me through its chromodomain (Clark and Elgin 1992; Nielsen *et al.* 2002; Fischle *et al.* 2003). HP1a is essential for centromeric satellite heterochromatin compaction and silencing (Lachner *et al.* 2001; Nakayama *et al.* 2001). Additionally, HP1 proteins contain a C-terminal chromo-shadow domain that contributes to dimerization, and a hinge region that binds RNA and promotes HP1 association with chromatin (Muchardt *et al.* 2002; Meehan *et al.* 2003; Maison and Almouzni 2004; Keller *et al.* 2012; Eissenberg and Elgin 2014). So far, five *C. elegans* proteins (Table 1) have been shown to recognize methylated H3K9, although only two, CEC-4 and LIN-61, were tested against a wide range of methylated substrates *in vitro*. Four of these, HPL-1, HPL-2, CEC-3/EAP-1, and CEC-4, contain chromodomains, whereas LIN-61 has four MBT motifs (Koester-Eiserfunke and Fischle 2011; Greer *et al.* 2014; Garrigues *et al.* 2015; Gonzalez-Sandoval *et al.* 2015).

HPL-1 and HPL-2 are the *C. elegans* orthologs of HP1, as they bear both a chromodomain and a chromoshadow domain, separated by a less well-conserved hinge region (Couteau *et al.* 2002). Mutants lacking *hpl-1* are phenotypically wild-type, whereas *hpl-2* mutants have pleiotropic defects including loss of repression of a heterochromatic reporter, slow growth, abnormal germ line development and sterility, somatic expression of germ line genes, and a synMuv phenotype (Couteau *et al.* 2002; Coustham *et al.* 2006; Schott *et al.* 2006, 2009; Simonet *et al.* 2007; Meister *et al.* 2011; Petrella *et al.* 2011; Towbin *et al.* 2012; McMurchy *et al.* 2017). While the two orthologs clearly have different functions, HPL-1 is partially redundant with HPL-2, as evidenced by enhanced sterility and growth defects of double mutants (Schott *et al.* 2006). HPL-1 and HPL-2 were also shown to promote fertility and vulval development synergistically with HIS-24, an H1 linker histone, which, when methylated on K14, has been shown by *in vitro* assays to be a target of HPL-1 recognition (Studencka *et al.* 2012).

HPL-1 and HPL-2 GFP fusion proteins are both widely expressed, yet the two fusions localize to different nuclear foci (Couteau *et al.* 2002; Schott *et al.* 2006). LIN-13, a multi-Zn-finger protein, was shown to be essential for the localization of HPL-2::GFP into foci and to physically interact with HPL-2, but had no effect on HPL-1::GFP (Coustham *et al.* 2006). HPL-1::GFP binds integrated transgene arrays, while HPL-2::GFP does not (Towbin *et al.* 2012). Additional studies of the HPL-2::GFP fusion showed that its foci became more peripheral upon disruption of euchromatic regulators such as the TIP60/NuA4 remodeling complex (Grant *et al.* 2010). Because it is unclear if the HPL-1::GFP and HPL-2::GFP fusions are fully functional, these localization studies should be repeated with HPL-1- and HPL-2-specific antibodies. Nonetheless, it is clear that these two HP1 proteins do not have identical binding sites or functions.

*In vitro*, HPL-2 can bind all three methylated forms of H3K9 as well as H3K27me3, but *in vivo* mapping by ChIP-chip in embryos showed that it binds primarily on the distal arms of autosomes in a pattern that correlates well with that of H3K9me1 and me2, but not with H3K9me3 or H3K27me3 (Garrigues *et al.* 2015). The HPL-2 signal is reduced, but not lost, in the *met-2set-25* double mutant, indicating that HPL-2 can associate with chromatin independently of H3K9 methylation (Garrigues *et al.* 2015), for example through binding another protein, methylation mark, or RNA. Genetic analyses also indicate that *hpl-2* has roles independent of H3K9 methylation, because *hpl-2* mutants have stronger sterility phenotypes at high temperature than *met-2set-25* mutants (Garrigues *et al.* 2015). Intriguingly, HPL-2 is found at many expressed genes, even in the distal arms of autosomes, and 94% of HPL-2-bound genes on chromosome arms also show binding by the nuclear envelope protein LEM-2 (Garrigues *et al.* 2015). This suggests that neither association with HPL-2 nor association with the nuclear envelope is sufficient to repress transcription.

CEC-3/EAP-1 is a chromodomain-containing protein that can bind to all methylated forms of H3K9 *in vitro*. Its association with germ line chromatin *in vivo* depends on MET-2, suggesting that it binds H3K9me1 or me2 (Greer *et al.* 2014). Loss of CEC-3 suppresses the transgenerational sterility observed in worms lacking the H3K4 demethylase SPR-5. Strains lacking SPR-5 accumulate H3K4me2 and lose H3K9me3 over multiple generations (Katz *et al.* 2009; Greer *et al.* 2014). Interestingly, *met-2* mutants also display transgenerational sterility, and the loss of MET-2 enhances *spr-5* sterility, while the loss of SET-25 has no effect (Katz *et al.* 2009). Given that *spr-5* mutants also accumulate high levels of H3K9me2 at several tested targets (Katz *et al.* 2009), it may be that an imbalance between H3K9me2 and H3K4me2 leads to transgenerational sterility in *spr-5* mutants. Interaction between these two methylation events is also suggested by the finding that loss of the H3K4 methyl transferase SET-2 rescues *hpl-2* somatic defects (Simonet *et al.* 2007).

LIN-61 is an MBT domain-containing protein that recognizes H3K9me2 and me3 (Harrison *et al.* 2007; Koester-Eiserfunke and Fischle 2011). Like *hpl-2*, *lin-61* is a synMuv gene, and mutants lacking LIN-61 have a reduced brood size (Harrison *et al.* 2007; Koester-Eiserfunke and Fischle 2011). LIN-61 has also been shown to form a complex with HPL-2 and LIN-13 (Wu *et al.* 2012). Both *hpl-2* and *lin-61* are hypersensitive to ionizing radiation (Johnson *et al.* 2013; McMurchy *et al.* 2017), contribute to the repression of transgene arrays (Towbin *et al.* 2012), and play roles in cell type-specific control of gene expression (Studencka *et al.* 2012; Zheng *et al.* 2013). Specifically, mutations in CEC-3, HPL-2, or LIN-61 alleviate the tissue-specific repression of *unc-4*, a transcription factor expressed in a subset of ventral cord (VC) neurons. Loss of the H3K9 methyl-transferase MET-2, HPL-2, LIN-61, or CEC-3 leads to the ectopic expression of *unc-4* in all VC neurons, while loss of the JMJD-2 histone demethylase reduces this unscheduled expression (Zheng *et al.* 2013). Such results underscore the partial redundancy found among H3K9me ligands (Schott *et al.* 2006; McMurchy *et al.* 2017), and suggests that H3K9 methylation is involved in the proper repression of some cell type-specific promoters (Zeller *et al.* 2016).

## CEC-4- and H3K9me-Mediated Anchoring at the Nuclear Periphery

**Figure 2:**
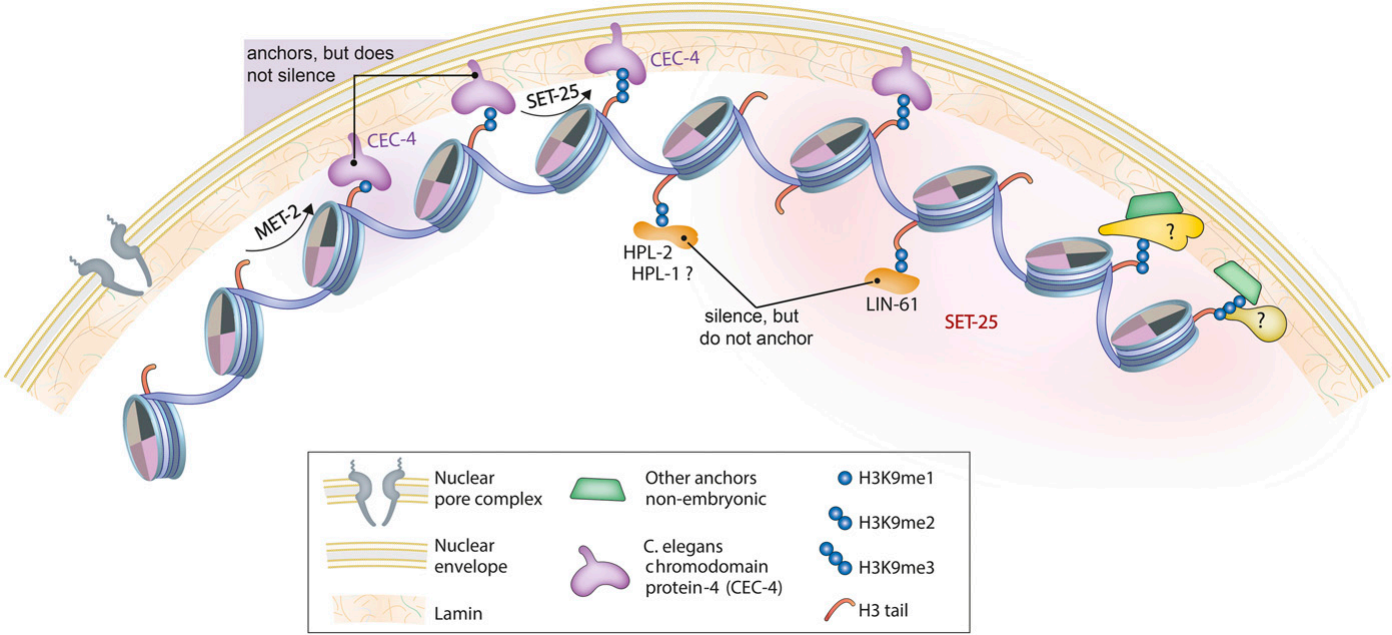
Histone H3K9 methylation triggers peripheral localization of chromatin independently of HPL-1/-2 or LIN-61 binding. In *C. elegans* early embryos, CEC-4 recognizes and binds H3K9 me1, me2, or me3 to mediate the anchoring of appropriately modified nucleosomes to the nuclear periphery, without necessarily repressing transcription (Gonzalez-Sandoval *et al.* 2015). The H3K9me ligands HPL-2 and LIN-61 mediate transcriptional repression by binding H3K9 methylation, but do not anchor chromatin. HPL-1 is associated with repressed chromatin, but its role in repression remains unclear. SET-25 colocalizes with heterochromatic transgene arrays bearing H3K9me3, and its activity, along with HPL-2 and LIN-61, leads to repression. MET-2 and/or SET-25 deposit H3K9me1 and me2, while only SET-25 deposits H3K9me3 in somatic cells (Towbin *et al.* 2012). Alternative anchors may be present in differentiated cells, although their identity is unknown.

CEC-4 is a worm-specific chromodomain protein necessary for perinuclear anchoring of heterochromatin (Gonzalez-Sandoval *et al.* 2015). It has a canonical HP1β-like chromodomain that recognizes histone H3 tails methylated on K9, but it lacks the chromoshadow motif (Gonzalez-Sandoval *et al.* 2015). Its C-terminal domain lacks all significant homology to other characterized chromatin-binding proteins, yet it is required for CEC-4 localization to the inner face of the nuclear envelope. Its perinuclear localization is recapitulated when CEC-4 is expressed as a fusion protein both in yeast and in worms, where it ensures the peripheral tethering of H3K9-methylated chromatin throughout early development (Gonzalez-Sandoval *et al.* 2015). The chromodomain of CEC-4 binds mono-, di-, and trimethylated H3K9 with affinities similar to mammalian HP1β, and with high specificity: out of 188 methylated histone peptides tested, only histone H3 tail peptides bearing K9 methylation, and, more weakly, H3K37me2, were bound. Because the latter modification has never been detected in *C. elegans*, the H3K9me is probably the most important ligand. Indeed, ablation of two conserved aromatic residues in the CEC-4 chromodomain was sufficient to compromise the tethering of heterochromatin at the nuclear envelope in *C. elegans* embryos (Gonzalez-Sandoval *et al.* 2015). This CEC-4-H3K9me interaction is the first chromodomain–ligand interaction shown to be necessary for chromatin positioning in the interphase nucleus in any species (Figure 2).

Although dependent on CEC-4, perinuclear heterochromatin tethering in embryos is independent of HPL-1, HPL-2, and LIN-61 and of H3K27me3 (Figure 2). Indeed, the tethering of large transgene arrays is independent of their transcriptional state, although it requires H3K9 methylation (Gonzalez-Sandoval *et al.* 2015). Consistently, the loss of CEC-4 had very little effect on gene expression under unchallenged growth conditions. Nonetheless, CEC-4 was needed for a full response to the ectopic expression of a cell fate regulator in pregastrulation embryos (Gonzalez-Sandoval *et al.* 2015). Following challenge by the induction of the myogenic transcription factor HLH-1 before gastrulation, wild-type embryos all arrest as differentiated masses of muscle cells. In the *cec-4* mutant, on the other hand, ∼25% of the embryos fail to arrest with a muscle-like phenotype, even though the induction of HLH-1 leads to muscle-specific protein expression. In other words, in the absence of CEC-4, other tissue lineages were not efficiently repressed, arguing that perinuclear anchorage helps restrict gene expression to that of the HLH-1-induced lineage (Gonzalez-Sandoval *et al.* 2015).

In another study, it was shown that CEC-4, H3K9 methyltransferases, and LEM-2 contribute to the condensed perinuclear status of the dosage-compensated X chromosomes in terminally differentiated postmitotic cells of adult animals (Snyder *et al.* 2016). This result suggests that nuclear organization, and specifically anchoring of chromosomal regions to the nuclear lamina, may affect dosage compensation. Nonetheless, during normal development, *cec-4* embryos develop into fertile adults.

## H3K9 Demethylases

Histone methyl marks are removed by demethylases that generally fall into two structural classes (Kooistra and Helin 2012). One class contains amine oxidases, of which LSD1 (a homolog of the *C. elegans* H3K4 demethylase SPR-5) is the founding member. These have been shown to be able to demethylate mono- and dimethylated lysine residues, but they are unable to act on trimethylated lysine. The second class contains the Jumonji C (JmjC) domain, which can act on all three methylation states.

*C. elegans* contains 13 JmjC domain-containing proteins (Klose *et al.* 2006), of which two (JMJD-2/JMJD2a and JMJD-1.2/CeKDM7a) are involved in H3K9 demethylation (Whetstine *et al.* 2006; Kleine-Kohlbrecher *et al.* 2010; Lin *et al.* 2010). IF of meiotic chromosomes showed that depletion of *jmjd-2* by RNAi led to increased H3K36me3 on the X chromosome and increased H3K9me3 on the autosomes (Whetstine *et al.* 2006). Reduction of JMJD-2 also led to replication stress, as indicated by a replication checkpoint-dependent increase in germ cell apoptosis, slow DNA replication fork progression (incorporation of Cy3-dUTP), and an accumulation of RAD-51 foci in the germ line, indicative of DNA breaks (Whetstine *et al.* 2006; Black *et al.* 2010). Interestingly, these germ line phenotypes could be rescued by deletion of the H3K9me reader HPL-2, suggesting that mistargeting of HPL-2 might be responsible for the phenotypes (Black *et al.* 2010). Loss of JMJD-2 would presumably lead to an accumulation of H3K9me, which may facilitate ectopic HPL-2 binding. Although H3K9me2 or me3 have been shown to correlate with late replication in other organisms (Schwaiger *et al.* 2010; Lubelsky *et al.* 2014), it is not yet clear if H3K9 methylation directly influences replication timing in *C. elegans*. Alternatively, perturbations in H3K9me levels may lead to replication stress that arrests replication (Zeller *et al.* 2016).

JMJD-1.2/CeKDM7a is a bispecific H3K9me2/H3K27me2 demethylase (Kleine-Kohlbrecher *et al.* 2010; Lin *et al.* 2010). Recombinant JMJD-1.2 has been shown to demethylate H3K9me2 and H3K27me2 *in vitro*, and *jmjd-1.2* mutants have increased levels of H3K9me2 and H3K27me2, by western blot analysis (Kleine-Kohlbrecher *et al.* 2010; Lin *et al.* 2010). ChIP of JMJD-1.2 has shown binding at H3K4me3-marked promoters, which are depleted for H3K9me2 and H3K27me2, and deletion of *jmjd-1.2* leads to decreased expression of a subset of tested targets (Lin *et al.* 2010). This may be due to the local acquisition of heterochromatic marks, although this has not yet been demonstrated. JMJD-1.2 appears to have a role in the nervous system, as a JMJD-1.2 transgene is predominantly expressed in neurons and *jmjd-1.2* mutants display movement defects (Kleine-Kohlbrecher *et al.* 2010).

## Phenotypes Caused by the Loss of H3K9 Methyltransferases or H3K9me Readers

*C. elegans* provides an opportunity to characterize the effects of a complete loss of H3K9 methylation during development of a multicellular organism, given that *met-2set-25* mutant embryos, larvae, and germ lines lack detectable H3K9 methylation, and are viable and fertile at 15 or 20° (Towbin *et al.* 2012; Ho *et al.* 2014; Garrigues *et al.* 2015; Zeller *et al.* 2016). Two recent studies investigated the consequences of an absence of H3K9 methylation or of interacting heterochromatin factors including H3K9me readers (Zeller *et al.* 2016; McMurchy *et al.* 2017). In adults, the heterochromatin factors studied were HPL-2 and LIN-61, the multi-zinc finger protein LIN-13 (Melendez and Greenwald 2000; Coustham *et al.* 2006; Harrison *et al.* 2007; Koester-Eiserfunke and Fischle 2011; Wu *et al.* 2012; Garrigues *et al.* 2015), and LET-418, an Mi-2 homolog that is part of the repressive NuRD (Nucleosome remodeling and histone deacetylase) and MEC (Mi-2 and MEP-1) complexes (von Zelewsky *et al.* 2000; Unhavaithaya *et al.* 2002; Passannante *et al.* 2010).

Unlike the situation in other metazoans, chromosome segregation is normal in the absence of H3K9 methylation (Zeller *et al.* 2016), possibly due to the holocentric nature of worm chromosomes. However, the loss of the MET-2 and SET-25 HMTs shares some phenotypes with the loss of the studied heterochromatin factors, such as the transcription of repetitive elements, an accumulation of DNA damage, reduced fertility, and increased germ cell apoptosis (Zeller *et al.* 2016; McMurchy *et al.* 2017). Not surprisingly, loss of the histone modification itself often showed more pronounced phenotypes than loss of a single reader.

Similar to other organisms, in *C. elegans* H3K9me2 and me3 are enriched on repetitive elements, with the majority of repetitive elements being marked by one or both modifications. Importantly, a detailed analysis of the two marks shows that they have different distributions: H3K9me2 is more significantly associated with a subset of DNA transposons and satellite repeats, while H3K9me3 was more prominent on retrotransposons and a second subset of DNA transposons (Zeller *et al.* 2016; McMurchy *et al.* 2017). With respect to genes, H3K9me3 is enriched on pseudogenes and silent cell type-specific genes, whereas H3K9me2 marks genes independently of their transcriptional activity (Ho *et al.* 2014; Garrigues *et al.* 2015; Zeller *et al.* 2016). H3K9me2, but not H3K9me3, is also associated with telomeres (McMurchy *et al.* 2017), although it is not required for their interaction with the nuclear envelope (Ferreira *et al.* 2013).

The genomic distributions of HPL-2, LIN-13, LIN-61, MET-2, and LET-418 are strikingly similar and highly correlated with H3K9me2, but not H3K9me3 (Garrigues *et al.* 2015; McMurchy *et al.* 2017). This begs the question of whether there are readers with a selective affinity for H3K9me3. While no direct binding studies have been reported to date, SET-25 colocalizes with repetitive arrays bearing H3K9me3, but not those with H3K9me2, suggesting that it might directly or indirectly associate with the me3 mark (Towbin *et al.* 2012). For HPL-1, its definitive binding specificity and endogenous genomic distribution remain to be determined. Preliminary evidence indicates that HPL-1 can recognize all three methylated states of H3K9 *in vitro* (W. Fischle, personal communication), and it bound methylated H3K23 in a peptide pull-down assay (Vandamme *et al.* 2015).

As mentioned above, a major function of H3K9me and heterochromatin proteins is transcriptional silencing of genes and repetitive elements. Previous work showed that deletion of the methyltransferases MET-2 and SET-25, or of HPL-2 or LIN-61, derepressed heterochromatic reporters (Towbin *et al.* 2012) and endogenous genes (Gonzalez-Sandoval *et al.* 2015). Mutants lacking H3K9me or any of the four studied heterochromatin proteins (HPL-2, LIN-61, LIN-13, or LET-418) derepressed genes and repetitive elements of all classes and families, including satellite repeats, simple repeats, and RNA and DNA transposons (Zeller *et al.* 2016; McMurchy *et al.* 2017). The majority of derepressed sequences are enriched for the relevant histone marks or chromatin factors in wild-type animals, suggesting that the effect is direct.

The observed derepression of repetitive elements is temperature-dependent, with many more repeat families being transcribed at 25° than at 20° (Zeller *et al.* 2016). This temperature dependence is particularly pronounced for tandem repeats. Temperature effects were also reported for the silencing of repetitive elements by the nuclear RNAi pathway (Ni *et al.* 2016), and for phenotypes of null alleles of other heterochromatin mutants and small RNA pathway factors (*e.g.*, Batista *et al.* 2008; Wang and Reinke 2008; Schott *et al.* 2009). Whether these reflect temperature-dependent hyperactivity of RNA polymerase II at 25°, a heat-stress response, or a sensitivity of other factors to heat is unknown. Interestingly, a recent report suggests that wild-type SET-25 activity may be temperature sensitive, and that its effect in silencing repetitive sequences can be transgenerationally inherited (Klosin *et al.* 2017). However, loss of *set-25* does not derepress endogenous micro- or mini-satellite repeats (J. Padeken and S. M. Gasser, unpublished results).

The detection of reproducible but low levels of repeat element transcription in *met-2set-25* mutant embryos argues for a broad misregulation of RNA polymerase II-mediated transcription caused by the loss of H3K9 methylation (J. Padeken, P. Zeller, and S. M. G., unpublished results). For simple repeats, transcriptional start sites are not yet mapped, but it is possible that cryptic transcription factor binding sites become exposed by the loss of repressive heterochromatin. Importantly, the loss of individual HMTs, *i.e.*, single *met-2* and *set-25* mutants, shows that different classes of repeats become expressed in the two mutants, consistent with their differential effects on H3K9me marks (J. Padeken, P. Zeller, and S. M. G., unpublished results).

The genes that carry H3K9 methylation in wild-type larvae and adults are generally pseudogenes or silent tissue-specific genes, and these too are derepressed in *met-2set-25* double mutants (Zeller *et al.* 2016; McMurchy *et al.* 2017). The expressed repetitive elements are highly enriched for full-length DNA transposases and LTR-containing elements derived from RNA transposons, which carry functional Pol II promoter sequences (McMurchy *et al.* 2017). Nonetheless, few elements marked by H3K9 methylation become expressed in its absence, suggesting that other mechanisms prevent repetitive element expression. For instance, loss of the nuclear RNAi component *nrde-2* leads to derepression of many elements not affected by the loss of H3K9 methylation (McMurchy *et al.* 2017). Nuclear RNA degradation mechanisms may be involved in repeat repression, as exosomes have been shown to play a role in heterochromatic silencing in other organisms (Shin *et al.* 2013; Sugiyama *et al.* 2016; Tucker *et al.* 2016). There may also be functional redundancy in repetitive element silencing among the heterochromatin factors studied, given that the mutants show redundancy in the promotion of fertility (McMurchy *et al.* 2017). In addition to transcriptional control, H3K9 methylation may prevent the movement of nonautonomous transposons or inhibit homologous recombination between repeats.

A striking phenotype associated with the loss of heterochromatin over repeat elements is the loss of genomic integrity. Increased germ line apoptosis in *met-2set-25* mutants was shown to be *cep-1/*p53-dependent and therefore linked to the DNA damage response (Zeller *et al.* 2016; McMurchy *et al.* 2017). Activation of the DNA damage response pathway contributes to the sterility of heterochromatin factor mutants, as sterility is partially suppressed by mutation of *cep-1*/p53, although this is not the case for *met-2set-25* (Zeller *et al.* 2016; McMurchy *et al.* 2017). Interestingly, resilencing of the MIRAGE1 DNA transposon by RNAi partially restored fertility in *hpl-2*, *let-418*, *and lin-13* mutants, potentially by suppressing expression of its transposase activity (McMurchy *et al.* 2017).

**Figure 3:**
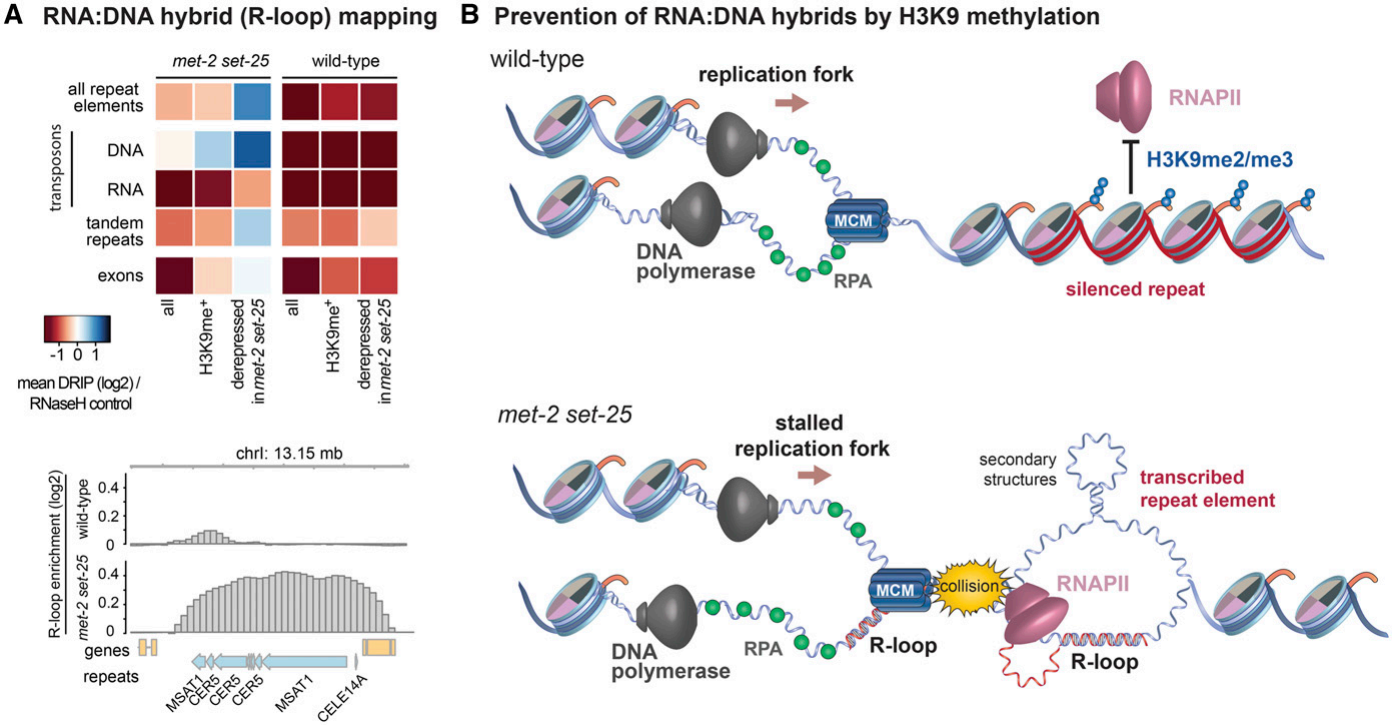
Larvae lacking MET-2 and SET-25 show an accumulation of RNA:DNA hybrids or R-loops at transcribed repeat elements. (A) Genome-wide distribution of R-loops determined by RNA:DNA immunoprecipitation (DRIP) with antibody S9.6, followed by deep sequencing of recovered DNA [see Zeller *et al.* (2016) for details]. Heat map of an S9.6 DRIP sequencing (DRIP-seq) experiment showing mean log2 enrichment over the corresponding RNaseH-treated controls. Loci are segregated based on the type of repeat element (vertical legend) and whether or not the sequences carry H3K9 methylation in wild-type cells (by chromatin immunoprecipitation analysis, labeled H3K9me+), or whether they were derepressed in *set-25 met-2* *vs.* wild-type embryos by RNA sequencing (horizontal legend). The lower panel is a DRIP-seq example showing the R-loop signal over a repeat element cluster. The immunoprecipitation signal is normalized to the input and the RNaseH control values were subtracted. (B) Transcribed repeat elements in H3K9me-deficient strains can exacerbate replication stress, provoking insertions and deletions in repetitive parts of the genome. This model suggests that the loss of H3K9me leads to R-loops, which allows the formation of secondary DNA structures that engender fork arrest, slippage, and breakage as forks deal with replication stress (Aguilera and Garcia-Muse 2012). These may perturb genome integrity, especially at heterochromatic repeats. Modified from Zeller *et al.* (2016). Chrl, Chromosome 1; RNAPII, RNA polymerase II.

The expression of repetitive sequences in *met-2set-25* mutant worms led to an increase in the formation of RNA:DNA hybrids or R-loops, a pathological annealing of RNA with DNA that generates a ssDNA loop where the RNA is bound (Zeller *et al.* 2016) (Figure 3). R-loops are the most common cause of replication fork-associated DNA damage (Aguilera and Garcia-Muse 2012). The RNA:DNA hybrids map specifically to the tandem repeats and DNA transposons that are derepressed by the loss of H3K9 methylation and, like transcription, the levels of RNA:DNA hybrids are enhanced at 25° *vs.* 20°, and are barely detectable at 15°, a temperature at which fertility defects are also suppressed (Zeller *et al.* 2016). The appearance of R-loops at repeat elements correlates with frequent small insertions and deletions at these sites in both somatic cells and the germ line, as detected by whole-genome sequencing (Zeller *et al.* 2016). Enhanced levels of Rad51 foci, indicative of spontaneous DNA damage, were observed in the germ lines of the *met-2set-25* double mutant, as in heterochromatic regions in *Drosophila* lacking the H3K9 HMT Su(var)3-9 (Peng and Karpen 2009). Importantly, *met-2set-25* mutant embryos and larvae are not hypersensitive to ionizing radiation, but are sensitive to replication stress induced by hydroxyurea (Zeller *et al.* 2016).

Some H3K9me readers play additional roles in DNA damage repair. Namely, LIN-61 mutants are sensitive to ionizing radiation and LIN-61 is needed for double-strand break repair by homologous recombination, but not by nonhomologous end-joining or single-strand annealing (Johnson *et al.* 2013). This leads to enhanced chromosome fragmentation and lower germ cell survival rates. Similarly, ionizing radiation in *hpl-2* mutants leads to increased checkpoint activation and oocyte chromosome fragmentation (McMurchy *et al.* 2017). Finally, RNAi-mediated reduction of the SPO-11 endonuclease partially rescues the fertility defect of K9me readers (McMurchy *et al.* 2017). The difference in hypersensitivities between H3K9me-deficient worms and hpl-2 or *lin-61* mutants, suggests that the roles of HPL-2 and LIN-61 in double-strand break repair may be independent of H3K9 methylation. Indeed, HPL-2 binds chromatin both in the presence and absence of H3K9 methylation (Garrigues *et al.* 2015), and in mammals the recruitment of HP1 proteins to sites of damage is H3K9me-independent (Luijsterburg *et al.* 2009; Baldeyron *et al.* 2011).

## PRC2/H3K27me3 and Interactions with MES-4/H3K36me3

The conserved PRC2 complex catalyzes methylation of lysine 27 of histone H3 and functions in the maintenance of transcriptional repression in metazoans (Steffen and Ringrose 2014; Piunti and Shilatifard 2016). The *C. elegans* PRC2-like complex is composed of MES-2, MES-3, and MES-6 (Bender *et al.* 2004). The SET domain protein MES-2, an ortholog of EZH2, provides H3K27 methylation activity. MES-6 is an ortholog of ESC/EED, and MES-3 is a novel protein.

The genes encoding PRC2 components were initially identified through genetic screens for genes required for germ cell development (Capowski *et al.* 1991). *mes-2*, *mes-3*, and *mes-6* mutants are maternal-effect sterile; homozygous mutants derived from heterozygous mothers are viable and fertile, but their progeny are sterile because germ cells die. The activities and functions of these genes are primarily germ line-specific. Somatic development of *mes-2*, *mes-3*, and *mes-6* mutants is overtly normal, although weak somatic defects in the expression of Hox genes has been observed (Capowski *et al.* 1991; Ross and Zarkower 2003). At least one somatically active H3K27 HMT exists because somatic H3K27 methylation is present in *mes-2* mutants, but it has not yet been identified (Bender *et al.* 2004).

In addition to components of PRC2, the *mes* screens also identified *mes-4*, which encodes a germ line-expressed H3K36 HMT with functions in both the germ line and in early embryos as a maternal product (Capowski *et al.* 1991; Bender *et al.* 2006). MES-4 is a nuclear receptor binding SET domain (NSD)-type HMT, that also bears PHD and post-SET domains, and methylates H3K36me in a transcription-independent manner. (Bender *et al.* 2006; Furuhashi and Kelly 2010; Rechtsteiner *et al.* 2010). In addition to MES-4, *C. elegans* also contains MET-1, an ortholog of the transcription-coupled H3K36 HMT SET-1 (Andersen and Horvitz 2007; Furuhashi and Kelly 2010; Rechtsteiner *et al.* 2010). The loss of MES-4 also results in phenotypes in somatic cells of L1 larvae derived from homozygous adults (D. Cabianca and S.M.G., unpublished results). Mass spectrometry of histones isolated from L1 larvae shows that the loss of MET-1 eliminates 95% of the H3K36me3, while the full complement of me1/me2 is retained. RNAi depletion of MES-4 in *met-1* mutants reduces H3K36me1/me2 and the residual H3K36me3 in an additive manner (D. Cabianca and S. M. Gasser, unpublished results).

Studies of PRC2 and *mes-4* mutants revealed an intimate relationship between H3K27 and H3K36 methylation. Genome-wide profiling in embryos and L3 larvae showed that H3K36me3 and H3K27me3 occupy mutually exclusive domains on autosomes, consistent with the finding that methylation of H3K36 inhibits EZH2 activity (Schmitges *et al.* 2011; Yuan *et al.* 2011; Gaydos *et al.* 2012). Additionally, the X chromosome has higher levels of H3K27me3 and lower levels of H3K36me3 than autosomes (Liu *et al.* 2011; Gaydos *et al.* 2012). Intriguingly, despite their marking different genomic regions, gene expression profiling in the germ line showed that loss of either MES-4 or PRC2 had similar consequences: reduced expression of germ line genes and increased expression of somatic and X chromosome genes (Gaydos *et al.* 2012). This was explained by showing that these two marking systems functionally antagonize each other. Loss of MES-4 causes spreading of H3K27me3 to germ line genes, and a concomitant reduction of H3K27me3 on somatic and X chromosome genes (Gaydos *et al.* 2012). Therefore, H3K27me3 marking by PRC2 and H3K36me3 by MES-4 cooperate to ensure correct gene expression in the germ line.

The chromatin modifications generated by MES-4 and PRC2 are transgenerationally inherited. MES-4 marks genes expressed in the germ line with H3K36me2/3, and then maternally contributed MES-4 maintains H3K36me2/3 marking of these genes in the early embryo independently of transcription, providing an epigenetic memory of germ line transcription to progeny (Furuhashi *et al.* 2010; Rechtsteiner *et al.* 2010). Similarly, H3K27 methylation generated by PRC2 in the germ line is inherited by progeny (Gaydos *et al.* 2014). Together, H3K27 methylation and maternal PRC2 components provide a memory of transcriptional repression (Gaydos *et al.* 2014).

**Figure 4:**
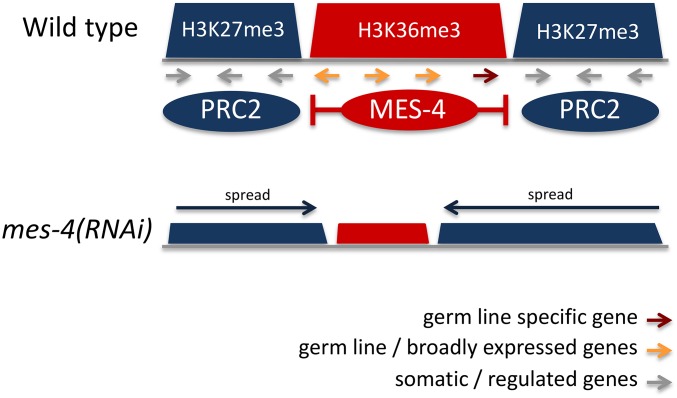
Functional antagonism between MES-4 and PRC2. The genome is organized into domains of genes expressed only in the germ line, or both in the germ line and broadly across cell types, and domains of genes with somatic cell and regulated expression. MES-4 marks genes transcribed in the germ line with H3K36me2/3. This includes germ line-specific genes (red) and broadly expressed genes (orange). A PRC2-like complex composed of MES-2, MES-3, and MES-6 marks somatic genes (gray) with H3K27me3. MES-4 inhibits PRC2: in *mes-4(RNAi)* embryos (derived from RNA interference-treated mothers), H3K27me3 marking spreads into regions previously occupied by H3K36me3.

A recent study used patterns of chromatin states in early embryos and L3 larvae to investigate domain properties of the *C. elegans* genome, defining two types of chromatin domain: active and regulated (Evans *et al.* 2016). It was found that active domains are associated with H3K36me3 and regulated domains with H3K27me3, which overlap the mutually exclusive patterns of these modifications noted by Gaydos *et al.* (2012). The domains separate genes of different types: active domains contain genes expressed in the germ line and broadly expressed across development and cell types, whereas regulated domains predominantly contain genes under spatial, temporal, or conditional control, lacking germ line expression. Regulated expression is consistent with the repressive role of H3K27me3 in facultative heterochromatin in other organisms. The locations of H3K36me3- and H3K27me3-marked domains in undifferentiated early embryonic cells were similar to those in differentiated L3 larvae, indicating that domain positions are a core property of the genome. The mechanism of domain definition is not yet understood, but appears to involve interactions between PRC2 and MES-4 (Gaydos *et al.* 2012; Evans *et al.* 2016) (Figure 4). Additionally, the finding that border regions between active and regulated domains contain long intergenic regions enriched for transcription factor binding suggests that transcriptional activity may play a role in defining the boundaries of active and regulated domains (Evans *et al.* 2016). A future challenge will be to investigate possible tissue-specific domain regulation by analyzing histone mark distribution in individual tissues rather than whole animals.

## PRC2 in Reprogramming and Maintenance of Cell Fate

PRC2 is important for the maintenance of a repressed state, and this may be due in part to inhibition of developmental plasticity. Early evidence for this in *C. elegans* came from a study showing that loss of *mes-2* in early (undifferentiated) embryos causes prolonged sensitivity to ectopic expression of developmental regulators that can cause cell fate transformations (Yuzyuk *et al.* 2009). This study also showed that loss of *mes-2* causes a change in chromosome conformation suggestive of a loss of compaction. Studies in germ cells similarly found that loss of PRC2 components makes germ cells susceptible to conversion to a somatic fate when challenged by expression of cell type-inducing transcription factors (Patel *et al.* 2012). Sensitivity to somatic conversion was also observed in response to increased Notch activity (Seelk *et al.* 2016). Notch appears to act by antagonizing PRC2 repression of numerous genes, including the H3K27me3 demethylase UTX-1. These studies support a role for PRC2 in preventing inappropriate responses to regulatory inputs, perhaps by increasing the barrier to response. Consistent with this role, the H3K27me2/3 demethylase JMJD-3.1 is necessary for a natural *C. elegans* transdifferentiation event, where a hindgut cell is transformed into a motor neuron (Zuryn *et al.* 2014).

## Chromatin Regulators and Small RNA Pathways

**Figure 5:**
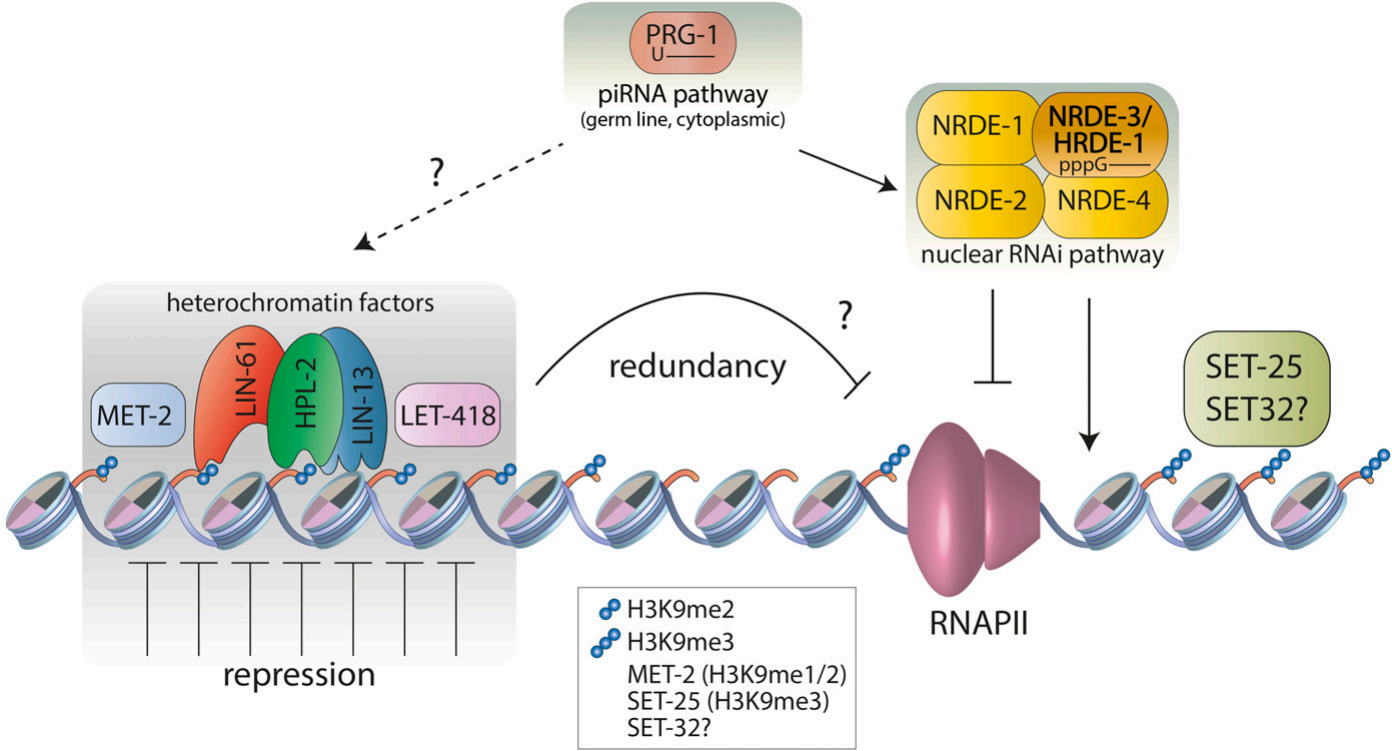
Heterochromatin proteins and small RNA pathways repress transcription of repetitive elements and genes. Repressed chromatin is marked by methylation of H3K9me2 and/or H3K9me3. The nuclear RNAi (Nrde) pathway uses small RNAs bound by argonaute proteins HRDE-1 (germ line) or NRDE-3 (soma) to target NRDE proteins to chromatin, leading to inhibition of transcription elongation and H3K9me3 marking, dependent on SET-25. Some H3K9me3 induced by exogenous application of RNAi is dependent on SET-32. Loss of NRDE function leads to derepression of repetitive elements (enriched for those derived from retrotransposons) and genes. The piRNA pathway, initiated in the germ line cytoplasm, targets genes and repetitive elements for repression by transcriptional and post-transcriptional mechanisms. The piRNA pathway engages the Nrde pathway for transcriptional silencing through the generation of small RNAs that bind HRDE-1. A set of heterochromatin factors are found together at many genomic locations and are expressed in the germ line and soma. They repress common elements by a mechanism that is not yet understood. Repetitive elements that require the heterochromatin proteins for repression (enriched for DNA transposons) largely differ from those requiring the Nrde pathway. This apparent difference appears to be at least partially due to functional redundancy between the Nrde pathway and heterochromatin factors, the nature of which is not yet known. piRNA, piwi-interacting RNA; RNAi, RNA interference; RNAPII, RNA polymerase II.

In addition to classical RNAi that carries out post-transcriptional silencing in the cytoplasm, *C. elegans* also has a nuclear RNAi pathway (called the Nrde pathway) that directs transcriptional silencing, whereby small RNAs provide sequence specificity that directs heterochromatin assembly at target loci and downregulation of RNA polymerase activity (Guang *et al.* 2008, 2010; Burkhart *et al.* 2011; Buckley *et al.* 2012; Gu *et al.* 2012; Mao *et al.* 2015). Although the precise mechanism of repression is still being worked out, a series of experiments showed that argonaute proteins bound to small interfering (siRNAs) (NRDE-3 in the soma or HRDE-1 in the germ line) recruit NRDE-1, NRDE-2, and NRDE-4 to target loci, leading to an accumulation of H3K9me3 in a MET-2- and SET-25-dependent manner, and the stalling of RNA polymerase II (Guang *et al.* 2008, 2010; Burkhart *et al.* 2011; Buckley *et al.* 2012; Gu *et al.* 2012; Mao *et al.* 2015) (Figure 5). Recently, SET-32 was also shown to affect HRDE-1-dependent H3K9 methylation (Kalinava *et al.* 2017; Spracklin *et al.* 2017), and MORC-1 has been implicated as a downstream effector needed to maintain H3K9me3 at HRDE-1 targets (Spracklin *et al.* 2017; Weiser *et al.* 2017). Additionally, it was found that MES-2-dependent H3K27me3 is also induced at Nrde targets (Mao *et al.* 2015), although the relationship between PRC2 and Nrde silencing is not yet known. The results support a model whereby the small RNA-targeted Nrde pathway represses transcription by triggering the generation of repressed chromatin. Interestingly, mutants affecting nuclear RNAi processes often show progressive sterility over generations (Table 2).

The Nrde pathway is also engaged by the piwi-interacting RNA (piRNA) pathway, a small RNA pathway active in the germ line, which is important for the repression of transposable elements as well as endogenous genes (Ashe *et al.* 2012; Bagijn *et al.* 2012; Lee *et al.* 2012) (Figure 5). piRNAs are nuclear-encoded 21-nucleotide RNAs (starting with a U) that are bound by the argonaute PRG-1 in the cytoplasm (Ruby *et al.* 2006; Batista *et al.* 2008; Das *et al.* 2008; Wang and Reinke 2008). These direct the generation of secondary siRNAs that become bound by the germ line Nrde argonaute, HRDE-1, to engage the Nrde pathway in transcriptional silencing (Ashe *et al.* 2012; Buckley *et al.* 2012; Luteijn *et al.* 2012). Silencing of a piRNA pathway reporter was shown to require the H3K9 HMTs SET-25, MET-2, and SET-32, as well as the heterochromatin proteins HPL-2, LIN-61, and LET-418 (Ashe *et al.* 2012; McMurchy *et al.* 2017). In adult worms, both *prg-1* mutants and mutants lacking these heterochromatin proteins derepress a common spectrum of repetitive elements, suggesting that these may work together in the germ line (McMurchy *et al.* 2017) (Figure 5).

Interestingly, loss of the Nrde pathway leads to the derepression of a different set of repetitive elements than loss of the piRNA pathway or of the above subset of heterochromatin factors (Kalinava *et al.* 2017; McMurchy *et al.* 2017). Retrotransposons are more affected in *nrde-2* mutants, whereas a bias for DNA transposons was observed for *prg-1* and heterochromatin mutants. Moreover, redundancy appears to underlie at least part of this apparent separation of function, because *nrde-2* genetically interacts with *let-418*, *lin-13*, and *hpl-2*. Additionally, *nrde-2*; *let-418* double mutants derepress many more repetitive elements than either single mutant (McMurchy *et al.* 2017) (Figure 5). Consistent with redundancy, we note that although the Nrde pathway induces H3K9 methylation, endogenous Nrde targets are still repressed in the absence of H3K9 methylation, indicating that H3K9 methylation is not necessary for repression by the Nrde pathway (Kalinava *et al.* 2017; McMurchy *et al.* 2017). The observed complexity and redundancy of silencing mechanisms underscores the importance of repeat element repression for genome stability.

## Perspectives

Heterochromatin fulfills many important functions in the genome of complex organisms. In this respect, *C. elegans* is no exception, as indicated by the diverse physiological defects of mutants lacking the enzymes that deposit heterochromatic marks or the proteins that recognize them. Repressive chromatin is essential for nuclear organization, genome domain structure, repression of repetitive elements, genome stability, and regulation of protein-coding genes. Cross talk and redundancy between the proteins and pathways involved has been observed, but these interactions are poorly understood. Furthermore, the specificity of most HMTs and histone mark readers remains to be determined, as does the nature and importance of the interactions observed between heterochromatin and small RNA interference mechanisms. Above all, the role of heterochromatin in controlling or shaping developmental programs is still an open question, and the genetics and rapid development of *C. elegans* are particularly useful for its investigation. As demonstrated in the past, *C. elegans* is an excellent organism for gene expression studies, given its genetic flexibility, rapid developmental time, and well-defined cell differentiation pathways.
